# Effects of different phenylcapsaicin doses on neuromuscular activity and mechanical performance in trained male subjects: a randomized, triple-blinded, crossover, placebo-controlled trial

**DOI:** 10.3389/fphys.2023.1215644

**Published:** 2023-08-02

**Authors:** Pablo Jiménez-Martínez, Juan Sánchez-Valdepeñas, Pedro J. Cornejo-Daza, Clara Cano-Castillo, Iván Asín-Izquierdo, Carlos Alix-Fages, Fernando Pareja-Blanco, Juan C. Colado

**Affiliations:** ^1^ Research Group in Prevention and Health in Exercise and Sport (PHES), University of Valencia, Valencia, Spain; ^2^ Life Pro Nutrition Research Center, INDIEX, Madrid, Spain; ^3^ ICEN Institute, Madrid, Spain; ^4^ Physical Performance and Sports Research Center, Universidad Pablo de Olavide, Sevilla, Spain; ^5^ Department of Biomedical Sciences, Faculty of Medicine and Health Sciences, University of Alcalá, Madrid, Spain; ^6^ Applied Biomechanics and Sport Technology Research Group, Autonomous University of Madrid, Madrid, Spain

**Keywords:** electromyography, neuromuscular physiology, resistance training, ergogenic aid, velocity-based training, strength endurance

## Abstract

**Objective:** This study aimed to examine the effects of phenylcapsaicin (PC) supplementation on strength performance and neuromuscular activity in young trained male subjects.

**Materials and methods:** A total of 25 trained subjects [full-squat (SQ) one repetition maximum (1RM) = 125.6 ± 21.0 kg] were enrolled in this randomized, triple-blinded, crossover, placebo-controlled trial. The subjects performed a first session and a post-24 h session for each condition. In the first session, the subjects ingested a high dose of PC (HD, 2.5 mg), a low dose (LD, 0.625 mg), or a placebo (PLA). Their performance in SQ was assessed under a 3% × 8 × 70% 1RM protocol in the first session. Their performances in countermovement jump (CMJ), SQ with 60% 1RM, and isometric squat were measured before and after the SQ protocol in both sessions. The neural activity of the vastus lateralis (VL) and vastus medialis (VM) was recorded via surface electromyography (EMG) and averaged in both sessions.

**Results:** Significant differences between the conditions were reported for lifting velocity, velocity loss, and the 60% load in dynamic SQ (*p* range = 0.02–0.04). Electrical changes were not identified for any outcome, although neural activity changed across time (*p* range ≤0.001–0.006). A significant condition × time effect was observed in CMJ compared to PLA (*p* ≤0.001) and LD (*p* ≤0.001). Intra-set analyses revealed higher velocities in HD compared to those in LD (*p* = 0.01) and PLA (*p* range = 0.004–0.008).

**Conclusion:** Therefore, PC may improve the strength performance and attenuate the mechanical fatigue induced by resistance training in SQ and CMJ exercises.

## Introduction

Human voluntary movement and force production are determined by the nervous system behavior (Alix-Fages et al., 2022b). During strenuous exercise, changes in neuronal circuitry (e.g., supraspinal structure excitability) lead to a decline in fatigue tolerance and sports performance (Alix-Fages et al., 2022a). In this regard, a heterogeneous group of substances known as capsaicinoids, which are found in chili peppers, has emerged as plausible ergogenic nervous system modulators (Jiménez-Martínez et al., 2022). Previously, researchers have focused on the impact of capsaicin (i.e., the main active principle of spicy peppers) on pain relief, weight loss, and performance (Arora et al., 2021; de Moura e Silva et al., 2021).

As a vanilloid-structured substance, capsaicin interacts with the transient receptor vanilloid 1 (TRPV1) (Hayman and Kam, 2008). TRPV1 are receptors related to afferent feedback from III and IV nerve fibers, a type of peripheral afferent fibers that are linked to the detection of pain and the development of central fatigue by affecting both supraspinal and spinal levels of the nervous system (Okano et al., 2015; Alix-Fages et al., 2022a). An exercise experience modulates the nervous system behavior, eliciting a higher tolerance to fatigue and discomfort in high-intensity efforts (i.e., near exhaustion) (Alix-Fages et al., 2022a). However, capsaicin and its analogs have been shown to modulate the mechanical responses to exercise during different intensities and under neural fatiguing conditions (Jiménez-Martínez et al., 2022). In this regard, TRPV1 agonists display their main physiological functions through reducing inflammatory hyperalgesia, downregulating voltage-activated calcium channels, and influencing thermoception (Baamonde et al., 2005; Hayman and Kam, 2008; Fattori et al., 2016). Accordingly, some discomfort-related responses to exercise, such as metabolite accumulation and calcium overload, are linked to III and IV afferent nerve fiber activity during exercise (Taylor et al., 2016; de Moura e Silva et al., 2021). Capsaicin supplementation is able to reduce the afferent signals of pain that are driven from the peripheral to the central nervous system, delaying the onset of fatigue in the neuromuscular junction (Taylor et al., 2016; Jiménez-Martínez et al., 2022). Consequently, the upregulation of TRPV1 leads to a decline in the rate of perceived exertion (RPE) and the perception of pain, as well as discomfort, during exercise (Jiménez-Martínez et al., 2022). For instance, recent evidence highlights that lumbar intrathecal fentanyl (afferent blockage) effectively attenuates the group III and IV afferent feedback during intermittent knee-extensor all-out exercise, resulting in improved physical performance and reduced RPE (Broxterman et al., 2018). Therefore, the increased firing of group III and IV muscle afferents, caused by mechanical forces and metabolite accumulation, has been well-documented to contribute to the development of central fatigue, affecting both spinal and supraspinal levels (Amann et al., 2011; Blain et al., 2016; Sidhu et al., 2017).

Recently, new analogs of capsaicin have been formulated to ensure higher bioavailability and gastrointestinal tolerance (Cross et al., 2020). Phenylcapsaicin (PC) is a synthetic analog of capsaicin composed of 98% of PC and its excipients (Turck et al., 2019). PC pharmacokinetics has shown a fast metabolism (i.e., 30 min) after administration (Turck et al., 2019). For these reasons, the PC ergogenic dose, although lower than that of traditional purified capsaicin, remains to be verified. On the other hand, capsaicin has demonstrated a positive influence on repetitions until failure, total volume load, and the rate of perceived exertion during resistance exercise interventions (de Freitas et al., 2018). Previous research has elucidated the effects of capsaicin on dynamic exercise (Jiménez-Martínez et al., 2022). In this regard, de Freitas et al. (2018) observed that 12 mg of capsaicin enhanced repetitions until exhaustion, total mass lifted, and the rate of perceived effort of four sets at 70% of maximum repetition (1RM) in the squat exercise (SQ) of a double-blinded, randomized, placebo-controlled intervention. However, these previous studies have not addressed different concerns that are still present in the literature. First, previous research studies are based on strength endurance protocols (Jiménez-Martínez et al., 2022), which may not be extrapolated to other types of exercise tasks, such as isometric contractions. In addition, the electromyographical mechanisms underlying the effects of capsaicin on resistance training performance and most of the mechanical outcomes used in the strength and conditioning field, which include the assessment of mechanical fatigue, have not been evaluated yet (de Moura e Silva et al., 2021). Moreover, in these previous studies, submaximal (i.e., not performed until failure) intensities were not assessed (Jiménez-Martínez et al., 2022), which reduces the real applicability of capsaicin on physical conditioning because the induction of excessive fatigue can disturb training adaptations (Pareja-Blanco et al., 2020a). Furthermore, most of the current research studies have not used objective measures of performance (e.g., infrared detection of jump height or linear velocity), which may hinder the estimate of the real impact of this substance on mechanical performance and fatigue (e.g., linear velocity loss) (Sánchez-Medina and González-Badillo, 2011).

In addition, as capsaicin may produce an analgesic effect, its impact on direct muscle force production and electromyographical outcomes is relevant. Currently, there are no previous data evaluating the influence of oral capsaicin on force production and neural responses during exercise. In addition, the electromyographical effects of capsaicin have only been evaluated for topical administration (Evans et al., 2021). In this study, topical capsaicin elicited significant changes in the motor unit recruitment pattern, which violated Henneman’s size principle in free-of-pain adults during voluntary trapezius and infraspinatus contractions (Evans et al., 2021). However, these neural responses have not been assessed during dynamic exercise after the ingestion of capsaicin, which makes it difficult to extrapolate this finding to sports performance. In addition, this issue must be highlighted because the neural strategies of the nervous system during exercise are reflected in force production during sports and in the induction of fatigue (Alix-Fages et al., 2022a). None of the previous studies has directly evaluated the effects of capsaicin on force production. Although this can be easily addressed with the use of a force platform (Piqueras-Sanchiz et al., 2021), the information obtained from this approach can be useful in determining whether this substance alters the amount, slope, or time of force production. On the other hand, the “desensitizer” effect of capsaicin on a sports task may lead to a higher degree of fatigue in the post-exercise window. Nevertheless, the effects of capsaicin on mechanical recovery outcomes have not been assessed yet (Jiménez-Martínez et al., 2022). This issue must be pointed out because an acute increase in fatigue could lead to a reduction in force production and sports performance during the subsequent training sessions or competitions (Pareja-Blanco et al., 2020b). Overall, the information about how capsaicin may modulate resistance training performance and fatigue, as well as the neural and mechanical mechanisms underlying these effects, is still lacking in current research studies.

Therefore, this study aims to examine the effects of two different doses of PC on fatigue and short-term mechanical responses, by measuring the isometric and dynamic performance, as well as neural activity, in CMJ and SQ exercises. For the first time, this study included the confluence of neural responses, dynamic and isometric exercise performances, and mechanical fatigue after capsaicinoid ingestion. It was hypothesized that PC may acutely increase velocity in the SQ exercise, neural excitability, and CMJ height. However, due to the improvements in performance, a higher degree of fatigue and a detrimental effect on recovery in post-test measurements were also expected.

## Materials and methods

### Experimental design

**FIGURE 1:**
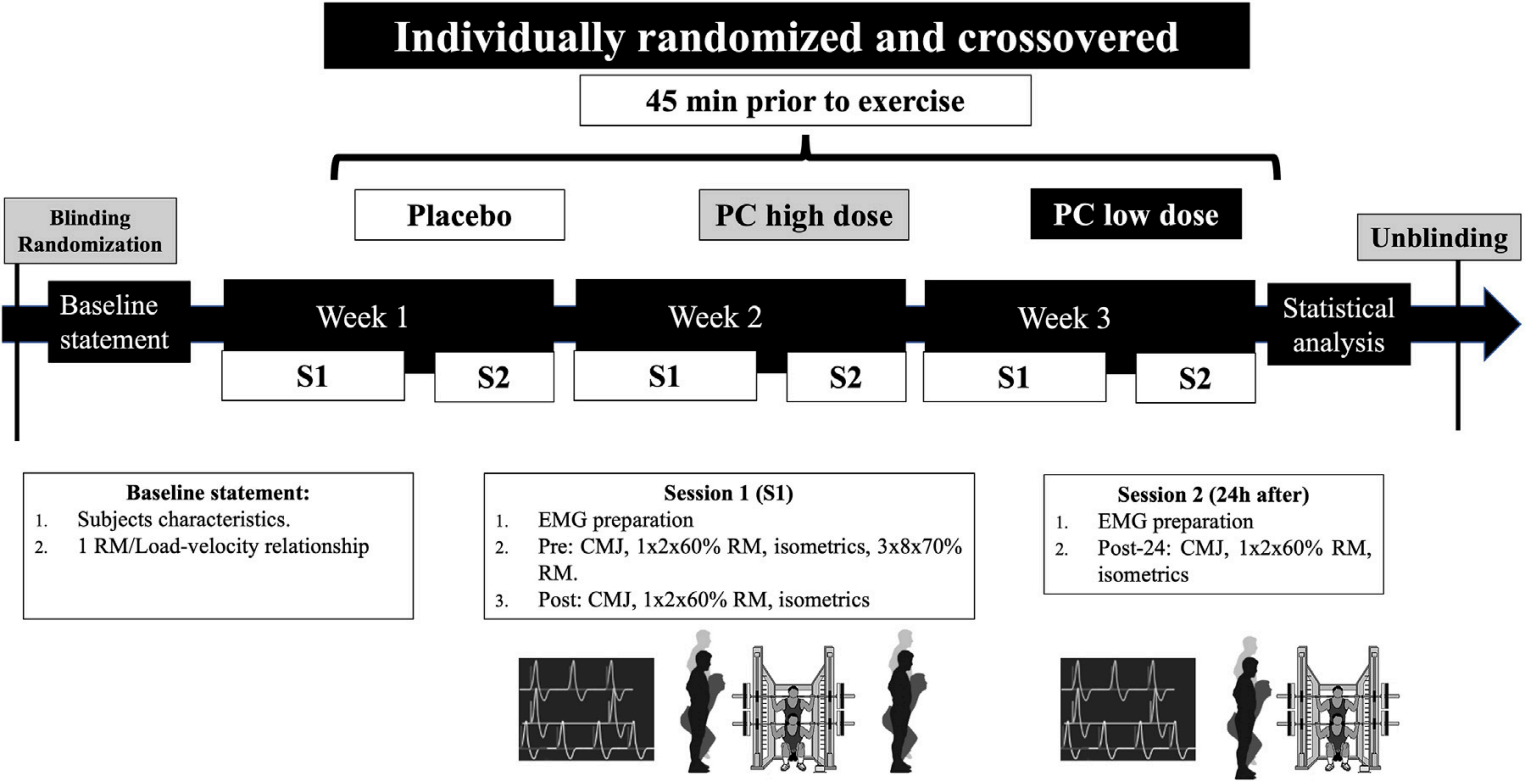
Timeline of the experimental protocol. PC, phenylcapsaicin; CMJ, countermovement jump; EMG, electromyography; 1RM, one-repetition maximum; S1/S2, session 1/2.

This study was conceived as a randomized, triple-blinded, crossover, placebo-controlled trial. Two weeks before the beginning of the study, the subjects were tested for anthropometrical measures (body mass and height), one-repetition maximum (1RM), and load–velocity relationship in SQ (see the Dynamic full-squat test section). Then, the subjects completed three experimental conditions, each one composed of a main session and a 24-h second session (post 24 h). The three experimental conditions were identical with the only difference in the supplement dose ingested. In addition, the subjects randomly ingested either a placebo (PLA) or a low (LD) or high (HD) dose of PC before the first weekly session. During the first weekly session, the subjects completed a SQ protocol that consisted of three sets of eight repetitions at 70% 1RM. Before (pre), immediately after (post), and 24 h after (post 24 h) the SQ protocol, a battery of tests was conducted to analyze the fatigue induced by each condition: CMJ, two SQ repetitions with 60% 1RM, and maximal isometric SQ at 90°, respectively. Since it is important to standardize temporalization to measure acute responses, the tests were conducted at the following time points: post-CMJ (1 min post-exercise), SQ with 60% 1RM (2 min post exercise), and isometric SQ (3 min post exercise). The electromyographical assessment of each session was recorded while the subjects were performing the SQ tests. The subjects performed each session at the same individual time of the day under stable environmental conditions (22°C–24°C and 55% humidity). The overall design of the study is depicted in Figure 1.

### Subjects

Sample size calculation was performed using G*POWER software (Heinrich-Heine-Universität Düsseldorf, Germany) with an alpha value of 0.05. Statistical power was fixed at 0.80 and the effect size at 0.60. Based on the total volume in the SQ exercise of the previous research (de Freitas et al., 2018), at least 21 subjects were required for this study. Finally, 25 healthy men (age = 21.7 ± 3.7 years, body mass = 77.4 ± 9.1 kg, height = 176.7 ± 7.2 cm, 1RM in SQ = 125.6 ± 21.0 kg, and 1RM normalized to body mass = 1.64 ± 0.22) with at least 2 years of experience in resistance training (range = 2–5 years) were enrolled in this study. If the subjects suffered from any cardiovascular, muscular, neurological, and/or metabolic disorder, they were directly excluded. Once the subjects were informed about the aim of the study, procedures, and possible risks, the subjects freely signed the informed consent sheet. The present research was approved by the Local Research Ethics Committee of Junta de Andalucía (Code: 0513-N-22) in accordance with the tenets of the Declaration of Helsinki. Each condition was established under the safety ranges proposed by the European Food Safety Authority (EFSA) expert panel (Turck et al., 2019).

The subjects were asked not to ingest stimulants (e.g., caffeine) or other ergogenic aids prior to each session, not to perform strenuous physical activity, and not to modify their dietary intake 2 days before the tests. One of the researchers reminded the subjects to maintain their normal dietary intake and not to train 2 days before the weekly testing sessions with a text message to each one separately. During 3 weeks of the study, two subjects withdrew from the study, one of them due to injury and the other because of missing a session.

### Procedures

#### Supplementation procedures

Supplements and placebo were prepared and packaged by a non-involved researcher in independent installations (Life Pro Nutrition industries, Madrid, Spain). To ensure triple blinding, each package was encoded with a number from one to three. The packages were not unblinded until a third-party researcher performed all the analyses. Packages and capsules were identical in appearance, color, and taste, and their content was only revealed after the independent researcher completed all the analyses. The capsule composition included the following: 1) PLA, a maltodextrin and excipient placebo with a red dye; 2) HD, 2.5 mg of PC (aXivite, Malmö, Sweden); and 3) LD, 0.625 mg of PC. Randomization and crossover were performed 2 weeks before the beginning of the study. To reduce possible bias, a third-party researcher assigned the subjects to each condition in the Research Randomizer website (www.randomizer.org). Each subject consumed one condition per week during the three total weeks of the study. PC doses or placebo was ingested 45 min prior to the first exercise session. Researchers encouraged the subjects to freely select a capsule from the daily assigned condition package. The capsules were taken with water under the supervision of at least one researcher. The randomized, counterbalanced, crossover sequences of each week of the study were eight HD, eight LD, and nine PLA in week 1; seven HD, 10 LD, and eight PLA in week 2; and 10 HD, seven LD, and eight PLA in week 3. This information was pooled after unblinding. The adverse effects were not reported for any of the supplementation conditions. Additionally, to ensure the absence of a maturation effect, order effect statistical analyses were also performed (Supplementary Tables S1–S3 of the Supplementary Material).

#### Electromyography

Before electromyography (EMG) recording, a researcher checked whether the subjects were properly shaved. Then, a black permanent marker was used to ensure consistency in the electrode position across conditions (Pareja-Blanco et al., 2020a). Surface EMG electrodes were placed over the vastus medialis (VM) and vastus lateralis (VL) of the right leg according to SENIAM criteria (Hermens et al., 2000) and the previous research of the field (Ortega-Becerra et al., 2021; Piqueras-Sanchiz et al., 2021). EMG signals were evaluated for 60% and 70% 1RM sets and isometric tests in both sessions. EMG signals were recorded continuously using a bipolar, parallel-bar surface electromyographic Wireless Trigno™ sensor (Delsys Inc., MA, United States) (r range = 0.92–0.99) (Poitras et al., 2019). Baseline noise was established at <5 µV peak-to-peak, and the sampling rate was 1926 Hz. The EMG system was set at an inter-electrode distance of 10 mm, common mode rejection ratio >80 dB, and bandwidth filter between 20 and 450 Hz ± 10% (Delsys Inc., MA, United States). Data were stored using EMGworks Acquisition software (Delsys Inc., MA, United States). For each measure, the median frequency (MDF) [ICC (95% CI): 0.95 (0.90–0.98) and CV: 5.3%] and root mean square (RMS) [ICC (95% CI): 0.95 (0.90–0.98) and CV: 7.4%] were individually calculated for VM and VL as excitatory muscle activity assessments and averaged for further analyses as described in the previous research (Kattla and Lowery, 2010; Piqueras-Sanchiz et al., 2021). All the outcomes measured were recorded for each repetition (over sliding windows of 500 ms with an overlap of 499 ms) and averaged for further analysis for dynamic and isometric tests. According to previous research, data were normalized under the daily maximal value of the first isometric signal (Piqueras-Sanchiz et al., 2021). Thus, EMG values were expressed as a percentage of the maximal daily value obtained (Hermens et al., 2000).

### Resistance exercise protocol

#### Warm-up

A standardized warm-up was performed 30 min after capsule ingestion in the first session and immediately after the subjects arrived at the laboratory in the second session. All the subjects were inspected regarding EMG marks before the warm-up. The general warm-up consisted of 5 min of continuous running at 9 km·h^−1^. Then, a specific warm-up was conducted before each test. The specific warm-up consisted of three sets of 10 repetitions of bodyweight SQs followed by three progressive CMJs and two maximal CMJs. Then, three SQ sets of two repetitions with 40%, 50%, and 60% 1RM were performed before the main SQ test.

#### Countermovement jump test

The CMJ height was determined using an infrared timing system (OptoJump Next, Microgate, Bolzano, Italy) (r = 0.99) (Glatthorn et al., 2011). The subjects were instructed to perform CMJs with their arms akimbo during eccentric and concentric phases. Accordingly, the CMJ technique was established at approximately 90° of knee flexion, followed by a maximal vertical jump. For each attempt, the landing was required to be in an upright position without bending the knees until the movement was completed. For each measurement, the subjects were required to perform two attempts with an interval of 10 s, and the mean value was calculated for further analyses [ICC (95% CI): 0.99 (0.97–0.99) and CV: 1.9%] (Piqueras-Sanchiz et al., 2021). If the jump height difference was greater than 2 cm between trials, a third measurement was made, and the two nearest values were averaged.

#### Dynamic full-squat test

An initial test with increasing loads was performed before the start of the study for the individual calculation of the 1RM and load–velocity relationship in the full-SQ exercises (González-Badillo et al., 2017). A Smith machine with no counterweight mechanism was used (Multipower Fitness Line, Peroga, Murcia, Spain). The mean propulsive velocity (MPV) was directly measured for each repetition using a linear velocity transducer (T-Force System, Ergotech, Murcia, Spain) (r = 0.99) (Sánchez-Medina and González-Badillo, 2011) attached perpendicularly to the barbell (González-Badillo and Sánchez-Medina, 2010). Although all the subjects were experienced in all the tests performed, as they had participated in at least two previous studies where these mechanical variables were assessed in the previous 3 months, the initial session was also used as familiarization. Accordingly, all the subjects performed isometric and CMJ tests after the progressive loading test.

Regarding the progressive loading test, the initial load used was 30 kg, and it was progressively increased to 10 kg until it reached a mean propulsive velocity of 0.50 m s^−1^ or lower. Then, the load was gradually increased (2.5–5.0 kg) until the repetition could not be completed. Three repetitions were completed for light loads (≥1.00 m s^−1^), two for medium loads (1.00–0.80 m s^−1^), and one for the heaviest loads (≤0.80 m s^−1^). Rest periods were set at 3 minutes for light and medium loads and 5 min for heavy loads. The load–velocity relationship was calculated with the best repetition of each attempt (i.e., highest MPV) (Piqueras-Sanchiz et al., 2021).

For the dynamic full-SQ evaluation, two SQ repetitions with 60% 1RM were performed. SQ was performed using a Smith machine (Fitness Line, Peroga, Murcia, Spain) with the subjects starting from the upright position with their knees and hips fully extended, parallel feet and their stance approximately shoulder-width apart, and the barbell resting across the back at the level of the acromion. Each subject descended in a continuous motion, until the top of their thighs was below the horizontal plane and the posterior thighs and shanks making contact with each other (∼35°–40° knee flexion), and then immediately reversed the motion and rose back to the upright position. Unlike the eccentric phase that was performed at a controlled mean velocity (∼0.50–0.65 m s^−1^), the subjects were encouraged to always perform the concentric phase of SQ at the maximal intended velocity (Pareja-Blanco et al., 2014). The mean propulsive values of velocity were acquired using a linear velocity transducer (T-Force System, Ergotech, Murcia, Spain) attached perpendicularly to the barbell. The highest values of each variable were recorded for further analyses. Resting between sets was set at 2 min.

#### Isometric squat test

The maximal isometric SQ test was performed at 90° of the knee flexion position (180° = full extension) for elucidating the effects of PC on the maximal isometric force (MIF) [ICC (95% CI): 0.99 (0.97–0.99) and CV: 3.4%] and the maximal rate of force development (RFDmax) [ICC (95% CI): 0.94 (0.86–0.97) and CV: 13.8%] (Piqueras-Sanchiz et al., 2021; Martinopoulou et al., 2022). For this purpose, a Smith machine with customizable height supports was equipped with an 80 × 80 cm dynamometric platform (FP-500, Ergotech, Murcia, Spain). The subjects were instructed to push with their legs against the floor of the platform as hard as possible after the cue “ready, set, go!” The subjects were required to execute two 5-s attempts separated by 1 min of rest per test. The external forces of each attempt were collected at a sampling rate of 1,000 Hz and processed using a specific software (T-Force System, Ergotech, Murcia, Spain) (r = 0.99) (Sánchez-Medina and González-Badillo, 2011). For the RFDmax assessment, the maximum slope in the force–time curve in 20-ms time intervals was selected. Furthermore, as RFD data were represented for different discriminable time gaps, RFD was calculated for the 0–50, 0–100, 0–150, 0–200, and 0–400 ms intervals. RFD and MIF outcomes were both averaged for further analyses. MIF was presented as the percentage of change from the pre-values. The specific warm-up consisted of two submaximal attempts at 70% and 90% of the maximal perceived effort.

### Full-squat protocol

The execution technique and setting have been described in the “Dynamic full-squat test” section. The SQ protocol consisted of three sets of eight repetitions with 70% 1RM with a 2-min rest period between sets. According to warm-up sets and individual load–velocity relationships, a 70% 1RM load was established daily for each subject. The MPV values for every repetition were recorded. The velocity loss (VLoss) induced within the set was calculated as the relative difference between the fastest repetition velocity and the last repetition velocity of each set (Sánchez-Medina and González-Badillo, 2011). The total volume load accumulated within the session was calculated using the following formula: absolute load lifted (kg) × total repetitions.

### Statistical analysis

The normal distribution of the variables and homoscedasticity were tested using the Shapiro–Wilk and Levene’s tests, respectively (*p* >0.05). A two-way repeated measures analysis of variance (ANOVA) (condition × time) with the Bonferroni *post hoc* test was used to explore the effect of the interventions (LD, HD, and PLA) across time on the magnitude of each dependent variable and to address the presence of an order effect. A one-way repeated measures ANOVA was used to compare the total volume load. The Greenhouse–Geisser correction was applied when Mauchly’s sphericity test was significant (*p* ≤0.05). Statistical analyses were performed using the software package SPSS (IBM SPSS version 25.0, Chicago, IL, United States). Statistical significance was established at *p* ≤0.05. To assess the magnitude of the differences, partial eta-squared values (ƞp^2^) were derived from ANOVA and were interpreted as low (<0.04), moderate (0.04–0.13), and large (>0.13). Bonferroni *post hoc* comparisons were used to evaluate pairwise differences. The effect size of *post hoc* comparisons was calculated by means of Cohen’s d, which was interpreted as a low (<0.50), moderate (0.50–0.79), or large effect (>0.80) (Cohen, 1988).

## Results

### Electromyography

The descriptive values and statistical comparisons for EMG outcomes are presented in Table 1. Two-way repeated measures ANOVAs did not reveal any condition × time interaction (*p* range <0.26–0.69). However, a significant time effect for RMS at 60% 1RM, MDF at isometric SQ, MDF at 60% 1RM, and MDF during the SQ protocol (*p* range <0.001–0.006) was observed. The *post hoc* Bonferroni test for time only revealed significant differences at 60% load RMS for 1/2 (*p* = 0.01; d = 1.11) and 2/3 (*p* = 0.006; d = 1.19) set comparisons.

**TABLE 1:** Comparison of electromyographical responses to the three different supplementation conditions using two-way repeated measures analysis of variance.

Variable	Time		Condition		ANOVA		
		PLA	LD	HD	Condition	Time	Condition × time
Isometric RMS (%)	Post	100.99 ± 23.72	101.31 ± 20.39	97.97 ± 35.00	F = 1.13; *p* = 0.33	F = 2.64; *p* = 0.11	F = 0.37; *p* = 0.69
	Post-24	105.59 ± 19.10	127.49 ± 34.42	107.19 ± 24.37	ƞp^2^ = 0.05	ƞp^2^ = 0.12	ƞp^2^ = 0.02
60% 1RM load RMS (%)	Pre	119.77 ± 24.11	125.20 ± 25.01	129.27 ± 39.11	F = 1.095; *p* = 0.35	F = 10.67; *p* <0.001*	F = 1.14; *p* = 0.34
	Post	102.82 ± 29.83	123.39 ± 40.81	102.84 ± 35.18	ƞp^2^ = 0.06	ƞp^2^ = 0.40	ƞp^2^ = 0.07
	Post-24	120.41 ± 31.13	132.88 ± 33.33	131.66 ± 26.60			
Isometric MDF (%)	Post	98.09 ± 10.34	95.87 ± 6.93	97.04 ± 8.23	F = 1.16; *p* = 0.32	F = 9.51; *p* = 0.006*	F = 0.49; *p* = 0.62
	Post-24	102.96 ± 15.81	95.95 ± 7.30	99.94 ± 8.14	ƞp^2^ = 0.05	ƞp^2^ = 0.32	ƞp^2^ = 0.02
60% 1RM load MDF (%)	Pre	88.22 ± 8.42	91.72 ± 9.65	89.81 ± 9.45	F = 1.20; *p* = 0.88	F = 3.52; *p* = 0.006*	F = 1.35; *p* = 0.26
	Post	87.50 ± 11.17	83.64 ± 6.36	88.98 ± 10.57	ƞp^2^ = 0.01	ƞp^2^ = 0.18	ƞp^2^ = 0.08
	Post-24	95.51 ± 16.42	89.16 ± 10.84	90.93 ± 11.88			
SQ protocol RMS (%)	Set 1	88.89 ± 8.27	89.47 ± 10.05	90.69 ± 10.80	F = 0.85; *p* = 0.36	F = 3.42; *p* = 0.07	F = 0.43; *p* = 0.52
	Set 2	87.13 ± 8.30	87.09 ± 9.85	89.23 ± 11.11	ƞp^2^ = 0.04	ƞp^2^ = 0.13	ƞp^2^ = 0.02
	Set 3	86.07 ± 8.80	85.91 ± 7.64	89.73 ± 10.77			
SQ protocol MDF (%)	Set 1	89.31 ± 8.74	90.55 ± 9.63	91.40 ± 9.93	F = 0.64; *p* = 0.54	F = 13.68; *p* <0.001*	F = 1.23; *p* = 0.29
	Set 2	87.06 ± 8.85	87.97 ± 9.83	90.19 ± 11.39	ƞp^2^ = 0.03	ƞp^2^ = 0.44	ƞp^2^ = 0.07
	Set 3	86.01 ± 8.32	86.14 ± 8.48	90.73 ± 11.06			

Mean ± standard deviation. PLA, placebo; HD, high dose; LD, low dose; RMS, root mean square; MDF, median frequency; VL, vastus lateralis; VM, vastus medialis. Post, post-exercise measure; post-24, 24 h post-exercise measure. Isometric: values obtained from the isometric squat test; 60% 1RM load: values obtained from two full-squat (SQ) repetitions against the 60% 1RM load; SQ protocol: values obtained from SQ protocol, i.e., from three SQ sets of eight repetitions with 70% 1RM load. * Significant difference (*p* ≤0.05).

### Countermovement jump test

The two-way repeated measures ANOVA revealed significant condition × time interactions for CMJ height (*p* <0.01) and a significant condition main effect (*p* <0.001) (Table 3). *Post hoc* Bonferroni tests showed that HD attained a higher CMJ height than PLA (*p* <0.001; d = 0.72) and LD (*p* <0.001; d = 0.37).

### Dynamic and isometric squat tests

Regarding the SQ test with 60% 1RM, the two-way repeated measures ANOVA did not reveal significant condition × time interactions (*p* range = 0.74–0.95). However, a significant time effect (*p* <0.001) and condition effect (*p* = 0.03) for MPV were observed (Table 3). Moreover, the two-way repeated measures ANOVA did not reveal significant condition × time interactions (*p* range = 0.09–0.89) or significant differences for time (*p*-range = 0.06–0.78) or condition effect (*p* range = 0.19–0.97) for any of the isometric outcomes (Table 2).

**TABLE 2:** Comparison of isometric mechanical responses to the three supplementation conditions using two-way repeated measures analysis of variance.

Variable	Time	Condition			ANOVA		
		PLA	LD	HD	Condition	Time	Condition × time
MIF (%)	Post	0.88 ± 0.13	0.90 ± 0.12	0.92 ± 0.13	F = 0.57; *p* = 0.57	F = 0.005; *p* = 0.95	F = 0.20; *p* = 0.81
	Post-24	0.93 ± 0.17	0.92 ± 0.19	0.95 ± 0.17	ƞp^2^ = 0.045	ƞp^2^ = 0.001	ƞp^2^ = 0.02
RFDmax (N·s^-1^)	Pre	4,562.8 ± 1,607.7	5,041.3 ± 1,676.7	4,363.2 ± 1,234.2	F = 0.03; *p* = 0.97	F = 0.30; *p* = 0.78	F = 1.46; *p* = 0.22
	Post	4,395.9 ± 1,689.3	3,935.8 ± 1,153.0	4,678.5 ± 1,658.9	ƞp^2^ = 0.07	ƞp^2^ = 0.12	ƞp^2^ = 0.06
	Post-24	4,894.2 ± 1,601.3	4,075.7 ± 1,337.7	4,752.2 ± 1,585.1			
RFD_0–50_ (N·s^-1^)	Pre	2,262.4 ± 1,189.2	2,765.4 ± 1,725.4	2,544.1 ± 1,150.3	F = 1.28; *p* = 0.29	F = 0.78; *p* = 0.46	F = 1.55; *p* = 0.19
	Post	2,467.7 ± 1,396.8	2,335.8 ± 1,408.5	2,375.3 ± 1,172.7	ƞp^2^ = 0.08	ƞp^2^ = 0.05	ƞp^2^ = 0.10
	Post-24	2,512.4 ± 1,332.5	2,006.3 ± 773.6	2,190.1 ± 1,249.6			
RFD_0–100_ (N·s^-1^)	Set 1	2,377.2 ± 1,325.0	2,921,4 ± 1,353.3	2,585.6 ± 1,998.9	F = 1.73; *p* = 0.19	F = 1.14; *p* = 0.33	F = 2.43; *p* = 0.10
	Set 2	2,039.6 ± 1,283.3	2,320.4 ± 1,229.5	1,968.5 ± 1,373.4	ƞp^2^ = 0.11	ƞp^2^ = 0.07	ƞp^2^ = 0.14
	Set 3	2,666.2 ± 1,432.5	2,129.3 ± 942.0	2,377.3 ± 1,196.7			
RFD_0–150_ (N·s^-1^)	Set 1	2,380.8 ± 1,655.6	2,958.4 ± 1,280.9	2,635.7 ± 1,075.2	F = 0.54; *p* = 0.58	F = 1.29; *p* = 0.28	F = 2.09; *p* = 0.09
	Set 2	2,670.8 ± 1,002.9	2,388.1 ± 1,184.6	2,129.5 ± 1,279.2	ƞp^2^ = 0.04	ƞp^2^ = 0.09	ƞp^2^ = 0.13
	Set 3	2,600.7 ± 1,330.5	2,260.5 ± 1,032.9	2,450.5 ± 1,275.4			
RFD_0–200_ (N·s^-1^)	Set 1	2,400.9 ± 1,322.2	2,697.3 ± 1,219.1	2,456.9 ± 1,052.1	F = 0.28; *p* = 0.76	F = 2.56; *p* = 0.10	F = 1.69; *p* = 0.16
	Set 2	2,384.1 ± 918.5	2,178.3 ± 1,101.1	1,973.3 ± 1,214.8	ƞp^2^ = 0.02	ƞp^2^ = 0.15	ƞp^2^ = 0.10
	Set 3	2,295.3 ± 1,430.7	2,067.3 ± 978.1	2,361.8 ± 1,214.6			
RFD_0–400_ (N·s^-1^)	Set 1	1,787.1 ± 974.8	1,764.9 ± 898.6	1,784.8 ± 809.4	F = 0.47; *p* = 0.62	F = 3.94; *p* = 0.06	F = 0.27; *p* = 0.89
	Set 2	1,652.5 ± 772.0	1,489.6 ± 733.9	1,521.3 ± 853,9	ƞp^2^ = 0.03	ƞp^2^ = 0.23	ƞp^2^ = 0.02
	Set 3	1,631.8 ± 1,045.1	1,610.3 ± 773.9	1,664.4 ± 912.0			

Mean ± standard deviation. PLA, placebo; HD, high dose; LD, low dose; MIF, maximal isometric force; RFDmax, maximal rate of force development; RFD_0–50_, rate of force development from the onset of force production to 50 ms; RFD_0–100_, rate of force development from the onset of force production to 100 ms; RFD_0–150_, rate of force development from the onset of force production to 150 ms; RFD_0–200_, rate of force development from the onset of force production to 200 ms; RFD_0–400_, rate of force development from the onset of force production to 400 ms.

### Full-squat protocol

**FIGURE 2:**
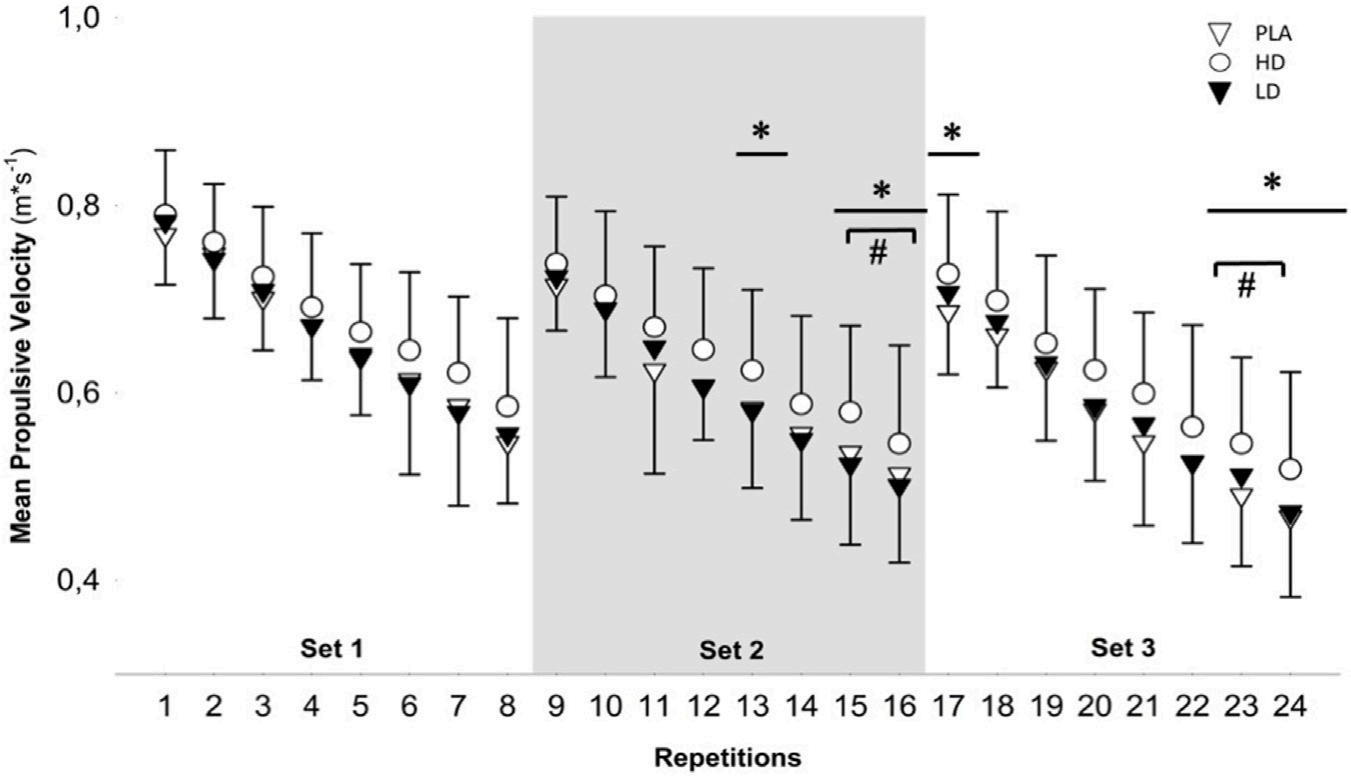
One-way repeated measures analysis of variance (ANOVA) of the intra-set comparisons of individual repetitions and repetitions across time for different supplementation conditions (PLA, HD, and LD). PLA, placebo; HD, high dose; LD, low dose (*p_*ANOVA*_ ≤0.05; #*p*_*Bonferroni*_ ≤0.05)

One-way repeated measures ANOVA reported no significant differences between conditions for total volume load (F = 1.09; *p* = 0.35). The two-way repeated measures ANOVA did not reveal significant condition × time interactions for any variable (*p* range = 0.12–0.98). However, it revealed significant time effects (*p* range = 0.001–0.008) for all the analyzed outcomes. Moreover, a significant condition effect was observed for 60% load (*p* = 0.03), MPVmean (*p* = 0.02), and VLoss (*p* = 0.04) (Table 3). The Bonferroni *post hoc* test revealed no significant differences for conditions (*p* range = 0.06–0.07). The *post hoc* Bonferroni test for time analyses revealed significant differences between sets 1 and 2 for all outcomes (*p* range <0.001 to 0.007; d range = 0.47–0.73). However, Bonferroni *post hoc* time analyses reported significant differences between sets 1 and 3 for MPV (*p* <0.001; d = 0.80). For comparisons between sets 2 and 3, all movement velocity outcomes reported significant time differences (*p* range <0.001 to 0.04; d range = 0.21–0.78). The one-way ANOVA for intra-set analysis revealed significant differences in repetitions 13, 15, 16, 17, 23, and 24 (*p* range = 0.03–0.04). The *post hoc* Bonferroni test reported significant differences between HD and PLA in repetition 23 (*p* = 0.01; d = 0.65) and for HD and LD in repetitions 15 (*p* = 0.008; d = 0.50) and 16 (*p* = 0.004; d = 0.46) (Figure 2).

**TABLE 3:** Comparison of mechanical responses to the three supplementation conditions using two-way repeated measures analysis of variance and comparison of the descriptive total volume of the squat protocol between the three supplementation conditions using one-way repeated measures ANOVA.

Variable	Time	Condition			ANOVA		
		PLA	LD	HD	Condition	Time	Condition × time
Total volume load (kg)		2,089.1 ± 357.6	2,014.6 ± 380.9	2,101.7 ± 352.9	F = 1.09; *p* = 0.35		
60% load MPV (m·s^−1^)	Pre	0.91 ± 0.07	0.93 ± 0.06	0.93 ± 0.06	F = 3.94; *p* = 0.03*	F = 50.89; *p* <0.001*	F = 0.48; *p* = 0.74
	Post	0.77 ± 0.09	0.80 ± 0.08	0.81 ± 0.12	ƞp^2^ = 0.19	ƞp^2^ = 0.75	ƞp^2^ = 0.03
	Post-24	0.89 ± 0.07	0.93 ± 0.09	0.91 ± 0.08			
MPVbest (m·s^−1^)	Set 1	0.77 ± 0.04	0.78 ± 0.05	0.79 ± 0.06	F = 1.40; *p* = 0.14	F = 65.53; *p* <0.001*	F = 0.01; *p* = 0.90
	Set 2	0.72 ± 0.04	0.72 ± 0.07	0.74 ± 0.07	ƞp^2^ = 0.09	ƞp^2^ = 0.73	ƞp^2^ = 0.013
	Set 3	0.70 ± 0.05	0.71 ± 0.07	0.73 ± 0.05			
MPVmean (m·s^−1^)	Set 1	0.65 ± 0.04	0.66 ± 0.07	0.68 ± 0.07	F = 4.14; *p* = 0.02*	F = 98.13; *p* <0.001*	F = 0.50; *p* = 0.77
	Set 2	0.60 ± 0.05	0.60 ± 0.07	0.64 ± 0.08	ƞp^2^ = 0.17	ƞp^2^ = 0.83	ƞp^2^ = 0.02
	Set 3	0.57 ± 0.06	0.58 ± 0.08	0.61 ± 0.08			
VLoss (%)	Set 1	29.4 ± 8.5	29.7 ± 10.9	26.6 ± 9.4	F = 3.73; *p* = 0.04*	F = 5.43; *p* = 0.008*	F = 0.11; *p* = 0.98
	Set 2	29.9 ± 11.8	30.9 ± 10.2	26.6 ± 11.7	ƞp^2^ = 0.14	ƞp^2^ = 0.21	ƞp^2^ = 0.006
	Set 3	34.2 ± 11.9	34.4 ± 12.2	29.8 ± 10.9			
CMJ height (cm)	Pre	39.94 ± 9.63	40.15 ± 9.61	40.18 ± 9.88	F = 97.10; *p* <0.001*	F = 2.82; *p* = 0.07	F = 3.48; *p* = 0.01*
	Post	32.92 ± 8.24	33.13 ± 8.29	34.73 ± 9.08	ƞp^2^ = 0.80	ƞp^2^ = 0.10	ƞp^2^ = 0.13
	Post-24	39.07 ± 9.12	40.02 ± 9.68	39.71 ± 9.71			

Mean ± standard deviation. PLA, placebo; HD, high dose; LD, low dose; MPV, mean propulsive velocity; VLoss, percentage of velocity loss during a set; MPVbest, the highest value of each set; MPVmean, the mean value of all repetitions conducted in each set; CMJ, countermovement jump. * Significant difference (*p* ≤0.05).

## Discussion

The objective of this study was to explore, for the first time, the neuromuscular and mechanical responses to a capsaicinoid supplement in dynamic and isometric exercises. The main findings of this research indicate that a LD or HD of PC does not modulate the electrical signals of quadriceps muscles compared to a PLA. However, the HD condition attained higher velocity values during the main SQ test than LD and PLA. Furthermore, the HD condition also exhibited higher CMJ values across time compared to PLA and LD. Supporting our initial hypothesis, PC may produce an ergogenic effect on SQ performance and may improve mechanical recovery outcomes (e.g., CMJ height and post-test lifting velocity). On the other hand, the initial hypothesis on a nervous system modulator effect was rejected according to the collected electrical signals. Therefore, a HD of PC supplementation may attenuate mechanical fatigue after a submaximal high-intensity session. These effects on fatigue outcomes may not be mediated by the traditional neural mechanisms proposed in the aforementioned literature (Jiménez-Martínez et al., 2022).

In the present study, changes in electrical signals were not detected for RMS or MDF between PLA and LD or HD. However, the protocol was effective, inducing fatigue due to the reduction in the intensity of electrical signals across the SQ sets. In this regard, an increase in RMS may be explained by a higher degree of motor unit recruitment, higher firing rates, or the recruitment of higher-threshold motor units when greater electrical outputs are needed (Bigland-Ritchie et al., 1986; Hunter et al., 2004). By contrast, lower MDF values are highly correlated with a decline in the force production from the fresh state due to impairments in the neural conduction velocity and discharge rates of motor units (Allison and Fujiwara, 2002). Thus, during and after high-intensity exercise, EMG can reflect the changes in neural strategies of the muscle related to the increment in the metabolic activity, hydrogen ion accumulation, and other physiological events inducing fatigue (Mendez-Villanueva et al., 2008). Contrary to the present findings, the previous research has documented that topical capsaicin administration may alter Henneman’s size principle (De Luca and Contessa, 2012), eliciting a greater number of motor units recruited in upper-limb isometric contractions (Evans et al., 2021). Nevertheless, the effects of an oral capsaicin supplement on electrical muscle signals have not been evaluated previously. In addition, although the mechanical performance was higher in the HD condition (i.e., lower velocity loss in all the sets), the null changes in electrical signals may be interpreted, bearing in mind that other physiological mechanisms may be presented in the ergogenic effect of PC supplementation (de Moura e Silva et al., 2021). Furthermore, if a direct neural mechanism would have appeared improving the neural output, HD may have attained higher RMS values during and in the post-squat window (Alix-Fages et al., 2022a), which suggests that PC does not provide an ergogenic effect on the acute recovery of the neural output. For this reason, PC may exert its main effects directly in the muscle junction upregulating the efflux of calcium channels and acetylcholine turnover without a direct effect on the neural raw output (de Moura e Silva et al., 2021).

Regarding mechanical outcomes, the present results have not displayed an ergogenic mechanical effect of PC on *post hoc* comparisons of velocity outcomes, although the CMJ height and intra-set lifting velocities were reported significant in the *post hoc* comparisons. However, as the velocity was higher in both HD and LD of PC, a plausible delaying effect on neuromuscular fatigue may have been presented (i.e., a lower velocity loss in all the sets and a higher CMJ height). Within this context, previous research has focused on the effects of capsaicin supplementation on strength endurance tasks performed until exhaustion (Jiménez-Martínez et al., 2022). For instance, a four sets at 70% 1RM until failure SQ protocol documented an ergogenic response to 12 mg of purified capsaicin in comparison to a placebo (de Freitas et al., 2018). In this study, the authors reported a higher number of repetitions until exhaustion and, consequently, a rise in the total mass lifted. However, the effects of capsaicin on mechanical fatigue were only approached by the inter-set analysis of the number of repetitions in the SQ exercise. Moreover, the effects of capsaicin on a non-exhaustive task after an SQ exercise have not been assessed previously (de Moura e Silva et al., 2021; Jiménez-Martínez et al., 2022). Accordingly, these aforementioned protocols may provide low applicability on the strength and conditioning or sports performance fields due to the high levels of fatigue induced (Pareja-Blanco et al., 2020b). Nevertheless, according to the current results and previous research, capsaicin may improve efforts performed close to muscular failure on account of the regulation of TRPV1 in the context of high degrees of metabolic fatigue during exercise (de Freitas et al., 2018; de Freitas et al., 2019), due to the responses of III and IV nerve afferent fibers (Taylor et al., 2016). For this reason, it could be hypothesized that the differences reported between the current study and the previous research may be explained by the fact that in the present study, the exercise volume was matched. This is corroborated by the growing gap reported between increasing MPVs in the intra-set analysis, where most of the significant differences were found in favor of the HD condition at the end of the last repetitions and mainly in the last set.

Concerning the influence of HD on mechanical recovery, the two dynamic indicators of fatigue (i.e., increasing MPV with 60% 1RM and CMJ height) exhibited higher values in the post-exercise window in favor of HD. According to the previous literature, SQ dynamic strength (Nuzzo et al., 2008) is highly correlated with the CMJ height (Bauer et al., 2019). Consequently, these effects on CMJ performance may have been mediated by the less mechanical fatigue accumulated according to the % of VLoss reported in the HD group. These findings are meaningful, as the reported improvements in the mechanical outcomes can be associated with a 2.5% enhancement of force production (Sánchez-Medina et al., 2017). Nonetheless, in both dynamic mechanical recovery variables, the post-24 h values returned close to the baseline, which suggests that this mechanical effect may be only considered during the first hours of the acute time course recovery window (Pareja-Blanco et al., 2019). Regarding isometric testing, as mentioned previously, the null effects of PC on MIF and RFD outcomes may be explained by the lower metabolic demands across the time required in isometric tasks (Wells et al., 2009). Accordingly, previous research did not report performance improvements from a low dose of capsaicin in knee-extension isokinetic exercises when the range of motion was restricted (Cross et al., 2020). Thus, PC’s TRPV1 activity may enhance resistance training during dynamic exercises but not in short-duration isometric tasks. Moreover, this rationale could also be the cause of the contrary results observed between dynamic fatigue and isometric fatigue in the present study, given that afferent III and IV nerve fibers mainly detect metabolic discomfort during exercise (Collins et al., 2018; Alix-Fages et al., 2022a). Within this context, exercise “perceived pain” could be explained as a manifestation of these nerve fiber firing, which is a biochemical target of PC supplementation, and leads to a lesser extent of efficiency in the neuromuscular junction, reducing (e.g., regulation of calcium overload) the velocity of crossbridge cycling, a mechanism directly involved in dynamic contractions (Collins et al., 2018; de Moura e Silva et al., 2021; Jiménez-Martínez et al., 2022). Additionally, the lack of a dose–response effect in most of the variables studied (i.e., isometric and electrical) may be explained by the existence of a threshold in TRPV1 peripheral activation, which is in line with the different doses of capsaicin employed in the previous literature (Jiménez-Martínez et al., 2022). In this sense, the current HD dose of PC may correspond to the most used evidenced ergogenic dose of capsaicin due to its near five-fold higher bioavailability (Turck et al., 2019; Framroze, 2022; Jiménez-Martínez et al., 2022; Jiménez-Martínez et al., 2023).

Collectively, an acute HD (i.e., 2.5 mg) of PC may reduce mechanical fatigue (i.e., higher CMJ height) after submaximal resistance exercise, as a consequence of a positive effect on the mechanical performance (i.e., higher MPV values and a lower % of VLoss) compared to LD and PLA. Therefore, PC may serve as a tool for reducing fatigue during high-volume resistance exercise workouts. Finally, a triple-blind, crossover, placebo-controlled design and the enrollment of trained subjects as described in previous research (de Freitas et al., 2018) were considered the important strengths of this current study. Nevertheless, some limitations may be remarkable. First, a nutritional follow-up of the subject’s diet and supplementation was not directly registered. Second, this study was conducted on trained men under laboratory conditions; for this reason, the present finding may be cautiously interpreted in other populations and environments. Moreover, bipolar surface EMG might not detect the contractile properties of the muscle in isolation. Accordingly, future research studies may add more accurate measures, such as high-density EMG, for the assessment of the neural effects of PC. Finally, the rate of perceived exertion or perceived pain was not evaluated in this study, which may be useful in future studies for the understanding of the sensitivity mechanisms underlying supplementation with capsaicin. On the other hand, it would be valuable to determine the impact of PC on protocols where exercise volumes were not matched. In addition, sets may be performed until exhaustion, and a force platform and linear transducer may be used.

## Conclusion

Acute PC ingestion may be considered an ergogenic aid (2.5 mg) for dynamic resistance exercise sessions when more than one exercise is performed. Consistently, mechanical fatigue after a submaximal exercise may be delayed and attenuated by PC. In addition, further research studies must deeply examine the existence of neural mechanisms underlying the capsaicinoid ergogenic impact.

## Data Availability

The raw data supporting the conclusion of this article will be made available by the authors, without undue reservation.
