# Supplementary material for: Effects of different phenylcapsaicin doses on neuromuscular activity and mechanical performance in trained male subjects: a randomized, triple-blinded, crossover, placebo-controlled trial
Source: Front Physiol. 2023 Aug 2;14:1215644. doi: 10.3389/fphys.2023.1215644 (PMC10433207; doi:10.3389/fphys.2023.1215644)
Supplement: Supplementary file 1 [file Table1.docx]

Supplementary Material

**Effects of different phenylcapsaicin doses on neuromuscular activity and mechanical performance in trained male subjects: A randomized, triple-blinded, crossover, placebo-controlled trial**

**Pablo Jiménez-Martínez^1,2,3^, Juan Sánchez-Valdepeñas^4^, Pedro J. Cornejo-Daza^4^, Clara Cano-Castillo^4^, Iván Asín-Izquierdo^4,5^, Carlos Alix-Fages^1,2,3,6^, Fernando Pareja-Blanco^4^, Juan C. Colado ^1*^**

^1^ Research Group in Prevention and Health in Exercise and Sport (PHES), University of Valencia, Valencia, Spain.

^2^ Life Pro Nutrition Research Center, INDIEX, Madrid, Spain.

^3^ ICEN institute, Madrid, Spain.

^4^ Physical Performance & Sports Research Center, Universidad Pablo de Olavide, Sevilla, Spain.

^5^ Department of Biomedical Sciences, Faculty of Medicine and Health Sciences, University of Alcalá, Madrid, Spain.

^6^ Applied Biomechanics and Sport Technology Research Group, Autonomous University of Madrid, Madrid, Spain**.**

*** Correspondence:** Juan C. Colado, Research Group in Prevention and Health in Exercise and Sport (PHES), Department of Physical Education and Sports University of Valencia C/ Gascó Oliag 3 46010, Valencia, Spain Tel.: 00 34 963 983 470 E-mail: juan.colado@uv.es

**Supplemenraty Material Table S1.** Two-way repeated measures analysis of variance (ANOVA) comparing the electromyographical responses during the three weeks of the study.

| Variable | Time |  | Condition | | ANOVA | | |
| --- | --- | --- | --- | --- | --- | --- | --- |
|  |  | W1 | W2 | W3 | Condition | Time | Condition × time |
| Isometric RMS (%) | Post | 92.03 ± 40.05 | 91.64 ± 27.55 | 103.58 ± 14.24 | F = 1.13; *p* = 0.33  ƞp² = 0.05 | F = 2.64; *p* = 0.11  ƞp² =0.12 | F = 0.37; *p* = 0.69  ƞp² = 0.02 |
|  | Post-24 | 127.47 ± 37.97 | 111.79 ± 27.40 | 108.27 ± 13.43 |  |  |  |
| 60% 1RM load RMS (%) | Pre  Post  Post 24 | 140.73 ± 45.66  104.26 ± 30.29  139.24 ± 34.65 | 116.06 ± 33.94  102.86 ± 39.23  116.64 ± 21.19 | 116.66 ± 20.14  110.45 ± 33.81  122.10 ± 33.18 | F = 0.71; *p* = 0.51  ƞp² = 0.06 | F = 13.72; *p =*<0.001*  ƞp² = 0.54 | F = 2.41; *p* = 0.10  ƞp² = 0.16 |
| Isometric MDF (%) | Post  Post-24 | 85.30 ± 18.13  99.44 ± 14.25 | 95.84 ± 10.23  102.59 ± 14.92 | 99.34 ± 12.56  96.86 ± 9.13 | F = 2.07; *p* = 0.14  ƞp² = 0.19 | F = 12.19; *p =*0.02*  ƞp² = 0.43 | F = 0.59; *p* = 0.55  ƞp² = 0.03 |
| 60% 1RM load MDF (%) | Pre  Post  Post-24 | 85.08 ± 11.46  88.08 ± 7.22  96.43 ± 14.45 | 93.14 ± 7.92  91.80 ± 12.33  93.92 ± 14.99 | 91.81 ± 9.69  90.94 ± 11.54  94.35 ± 13.73 | F = 0.35; *p* = 0.70  ƞp² = 0.02 | F = 2.10; *p* = 0.13  ƞp² = 0.12 | F = 0.41; *p* = 0.80  ƞp² = 0.25 |
| SQ protocol RMS (%) | Set 1  Set 2  Set 3 | 90.44 ± 8.19  88.46 ± 8.18  84.21 ± 7.63 | 92.12 ± 8.99  89.09 ± 6.55  89.54 ± 7.68 | 92.80 ± 8.65  91.58 ± 9.21  88.65 ± 8.86 | F = 0.43; *p* = 0.09  ƞp² = 0.21 | F = 5.54; *p* = 0.06  ƞp² = 0.22 | F = 0.40; *p* = 0.53  ƞp² = 0.02 |
| SQ protocol MDF (%) | Set 1  Set 2  Set 3 | 87.05 ± 7.43  84.65 ± 8.19  84.18 ± 7.54 | 82.97 ± 14.22  82.06 ± 14.13  89.59 ± 7.90 | 93.80 ± 9.50  87.58 ± 9.21  87.64 ± 8.84 | F = 8.15; *p* = 0.05  ƞp² = 0.35 | F = 0.10; *p* = 0.75  ƞp² = 0.006 | F = 1.13; *p* = 0.32  ƞp² = 0.06 |

Mean ± standard deviation. W1/2/3, Week 1/2/3; RMS, Root mean square; MDF; Median frequency; VL, Vastus lateralis; VM, Vastus medialis. Post: post-exercise measure; Post24: 24-hours post-exercise measure. Isometric: values obtained from the isometric squat test; 60% 1RM load: values obtained from the 2 full-squat (SQ) repetitions against the 60% 1RM load; SQ protocol; values obtained from the SQ protocol, i.e., from 3 SQ sets of 8 repetitions with 70% 1RM load. * Significant difference (*p* ≤ 0.05).

**Supplementary Material Table S2.** Two-way repeated measures analysis of variance (ANOVA) comparing the isometric mechanical responses during the three weeks of the study.

| Variable | Time |  | | Condition | | ANOVA | | |
| --- | --- | --- | --- | --- | --- | --- | --- | --- |
|  |  | W1 | W2 | | W3 | Condition | Time | Condition × time |
| MIF (%) | Post | 0.97 ± 0.36 | 0.84 ± 0.31 | | 1.01 ± 0.52 | F = 0.61; *p* = 0.54  ƞp² = 0.03 | F = 0.03; *p* = 0.87  ƞp² = 0.002 | F = 0.64, *p* = 0.53  ƞp² = 0.40 |
|  | Post-24 | 0.99 ± 0.42 | 1.05 ± 0.53 | | 1.04 ± 0.55 |  |  |  |
| RFDmax (N·s^-1^) | Pre | 4,088.15 ± 2,5244 | 3,619.25 ± 1,025.96 | | 4,296.55 ± 1,860.92 | F = 0.18; *p* = 0.83  ƞp² = 0.12 | F = 1.16; *p* = 0.32  ƞp² = 0.07 | F = 1.61; *p* = 1.83  ƞp² = 0.97 |
|  | Post | 4,020.1 ± 1,576.83 | 3,617.85 ± 1,626.29 | | 4,370.40 ± 1,537.39 |  |  |  |
|  | Post-24 | 3,961.85 ± 1,025.96 | 3,426.15 ± 1,654.14 | | 3,481.20 ± 1,444.67 |  |  |  |
| RFD_0-50_ (N·s^-1^) | Pre | 1,786.70 ± 696.73 | 2,330.30 ± 1,141.39 | | 2,201.90 ± 1,154.20 | F = 0.37; *p* = 0.56  ƞp² = 0.02 | F = 0.82; *p* = 0.39  ƞp² = 0.05 | F = 0.09; *p* = 0.92  ƞp² = 0.01 |
|  | Post | 1,920.20 ± 758.24 | 1,791.20 ± 613.76 | | 1,546.80 ± 902.10 |  |  |  |
|  | Post-24 | 1,756.30 ± 864.59 | 2,205.90 ± 885.51 | | 1,554.60 ± 896.05 |  |  |  |
| RFD_0-100_ (N·s^-1^) | Set 1 | 1,839.25 ± 1,104.88 | 2,552.15 ±1,367.38 | | 2,038.90 ± 1,014.47 | F = 1.14; *p* = 0.33  ƞp² = 0.08 | F = 1.73; *p* = 0.19  ƞp² = 0.11 | F = 2.43; *p* = 0.06  ƞp² = 0.14 |
|  | Set 2 | 2,125.15 ± 861.33 | 1,537. 55 ± 672.12 | | 2,108.80 ± 966.11 |  |  |  |
|  | Set 3 | 2,385.23 ± 1,104.54 | 1,822.70 ± 957.01 | | 1,751.80 ± 822.87 |  |  |  |
| RFD_0-150_ (N·s^-1^) | Set 1 | 1,768.45 ± 1,556.97 | 2,402.06 ± 984.14 | | 2,050.56 ± 1,084.55 | F = 1.29; *p* = 2.89  ƞp² = 0.09 | F = 0.55; *p* = 0.59  ƞp² = 0.37 | F = 2.10; *p* = 0.10  ƞp² = 0.13 |
|  | Set 2 | 2,232.27 ± 836.49 | 1,610.05 ± 870.09 | | 2.156,82 ± 1,077.26 |  |  |  |
|  | Set 3 | 2,471.23 ± 1,200.71 | 1,925.40 ± 966.10 | | 1,945.06 ± 1,034.40 |  |  |  |
| RFD_0-200_ (N·s^-1^) | Set 1 | 1,357.10 ± 841.20 | 2,330.30 ± 1,441.39 | | 2,201.40 ± 1,1451.64 | F = 2.23; *p* = 0.15  ƞp² = 0.14 | F = 0.05; *p* = 0.94  ƞp² = 0.001 | F = 7.13; *p* = 0.72  ƞp² = 0.21 |
|  | Set 2 | 1,345.57 ± 731.05 | 1,791.20 ± 714.24 | | 1,546.80 ± 1,554.33 |  |  |  |
|  | Set 3 | 1,363.82 ± 841.17 | 1,905.90 ± 885.12 | | 1,554.60 ± 896.07 |  |  |  |
| RFD_0-400_ (N·s^-1^) | Set 1 | 1,939.25 ± 1,204.52 | 2,140.14 ± 1,217.30 | | 1,450.51 ± 819.20 | F = 1.60; *p* = 0.20  ƞp² = 0.09 | F = 0.28; *p* = 0.79  ƞp² = 0.02 | F = 0.90; *p* = 0.46  ƞp² = 0.05 |
|  | Set 2 | 1,743.15 ± 1,061.42 | 1,435.85 ± 974.19 | | 1,491.32 ± 893,90 |  |  |  |
|  | Set 3 | 2,085.46 ± 1,453.10 | 1,923.82 ± 947.24 | | 1,3989.67 ± 818.05 |  |  |  |

Mean ± standard deviation. W1/2/3, Week 1/2/3; MIF; Maximal isometric force; RFDmax, maximal rate of force development; RFD0-50: rate of force development from the onset of force production to 50 ms; RFD0-100: rate of force development from the onset of force production to 100 ms; RFD0-150: rate of force development from the onset of force production to 150 ms; RFD0-200: rate of force development from the onset of force production to 200 ms; RFD0-400: rate of force development from the onset of force production to 400 ms.

**Supplementary Material Table S3.** Two-way repeated measures analysis of variance (ANOVA) comparing the mechanical responses during the three weeks of the study.

| Variable | Time |  | | Condition | | ANOVA | | |
| --- | --- | --- | --- | --- | --- | --- | --- | --- |
|  |  | W1 | W2 | | W3 | Condition | Time | Condition × time |
| 60% load MPV (m·s^-1^) | Pre | 0.92 ± 0.06 | 0.90 ± 0.08 | | 0.90 ± 0.06 | F = 0.14; *p* = 0.87  ƞp² = 0.08 | F = 62.98; *p*<0.001*  ƞp² = 0.78 | F = 0.88; *p* = 0.48  ƞp² = 0.50 |
|  | Post | 0.76 ± 0.10 | 0.78 ± 0.11 | | 0.75 ± 0.11 |  |  |  |
|  | Post-24 | 0.90 ± 0.09 | 0.89 ± 0.09 | | 0.89 ± 0.09 |  |  |  |
| MPV best (m·s^-1^) | Set 1 | 0.77± 0.04 | 0.74 ± 0.06 | | 0.76 ± 0.04 | F = 1.51; *p* = 0.23  ƞp² = 0.08 | F = 47.06; *p* <0.001*  ƞp² = 0.74 | F = 0.79; *p* = 0.53  ƞp² = 0.05 |
|  | Set 2 | 0.71 ± 0.05 | 0.69 ± 0.06 | | 0.70 ± 0.04 |  |  |  |
|  | Set 3 | 0.70 ± 0.05 | 0.69 ± 0.07 | | 0.69 ± 0.06 |  |  |  |
| MPV mean (m·s^-1^) | Set 1 | 0.66 ± 0.04 | 0.63 ± 0.07 | | 0.66 ± 0.08 | F = 2.05; *p* = 0.14  ƞp² = 0.10 | F = 66.47; *p* <0.001*  ƞp² = 0.80 | F = 1.39; *p* = 0.24  ƞp² = 0.07 |
|  | Set 2 | 0.62 ± 0.04 | 0.59 ± 0.08 | | 0.60 ± 0.08 |  |  |  |
|  | Set 3 | 0.59 ± 0.07 | 0.57 ± 0.08 | | 0.59 ± 0.08 |  |  |  |
| VLoss (%) | Set 1 | 28.6 ± 7.5 | 27.6 ± 9.0 | | 27.5 ± 11-2 | F = 2.40; *p* = 0.10  ƞp² = 0.12 | F = 4.37; *p* = 0.02*  ƞp² = 0.21 | F = 1.09; *p* = 0.37  ƞp² = 0.06 |
|  | Set 2 | 24.8 ± 11.1 | 26.8 ± 12.6 | | 26.14 ± 12.5 |  |  |  |
|  | Set 3 | 30.7 ± 13.44 | 31.2 ± 11.7 | | 28.12 ± 10.14 |  |  |  |
| CMJ height (cm) | Pre | 37.82 ± 9.33 | 36.71 ± 6.67 | | 35.19 ± 7.31 | F = 0.69; *p =* 0.50  ƞp² = 0.04 | F = 1.95; *p* = 1.57  ƞp² = 0.10 | F = 0.81; *p* = 0.51  ƞp² = 0.05 |
|  | Post | 36.03 ± 10.26 | 37.46 ± 7.31 | | 35.14 ± 10.60 |  |  |  |
|  | Post-24 | 37.17 ± 9.50 | 36.80 ± 7.05 | | 35.60 ± 10.96 |  |  |  |

Mean ± standard deviation. W1/2/3, Week 1/2/3; Mean propulsive velocity; VLoss, Percentage of velocity loss during a set; Best, the highest value of each set; Mean, the mean value of all repetitions conducted in each set; CMJ, Countermovement jump. * Significant difference (*p* ≤ 0.05)

**
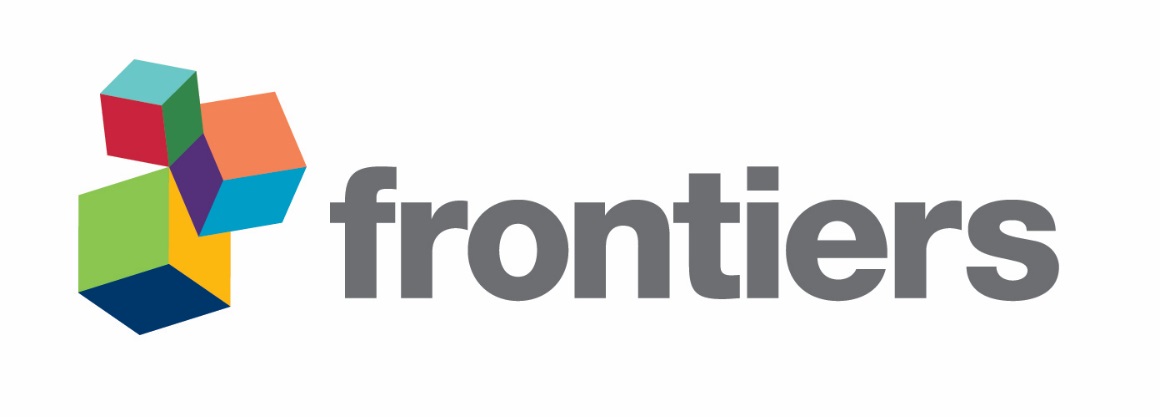
**
